# Monolithic and Minimally Veneered Zirconia Complications as Implant-Supported Restorative Material: A Retrospective Clinical Study up to 5 Years

**DOI:** 10.1155/2020/8821068

**Published:** 2020-10-15

**Authors:** Markel Diéguez-Pereira, David Chávarri-Prado, Alejandro Estrada-Martínez, Esteban Pérez-Pevida, Aritza Brizuela-Velasco

**Affiliations:** ^1^Department of Surgery and Medical-Surgical Specialties, Faculty of Medicine, University of Oviedo, Av. Julián Clavería 6, 33006 Oviedo, Spain; ^2^Department of Surgery, Faculty of Medicine, University of Salamanca, Campus Miguel de Unamuno, 37007 Salamanca, Spain; ^3^Faculty of Health Sciences, Miguel de Cervantes European University, C/Padre Julio Chevalier 2, 47012 Valladolid, Spain

## Abstract

**Objective:**

Long-term clinical data on the success and complication rates of monolithic or minimally veneered zirconia implant-supported restorations are lacking. Hence, the purpose of this retrospective clinical study was to analyze the complications of monolithic or partially veneered zirconia implant-supported restorations up to 5 years follow-up. *Material and Methods*. Single crowns, bridges, and full-arch rehabilitations were included. The selection process was achieved by reviewing data from the prosthetic laboratory and excluding cases in which zirconium and full-ceramic coating restorations were used. A total of 154 restorations were included (82 monolithic and 72 with buccal ceramic stratification). All the complications encountered, and the solutions applied, were explained.

**Results:**

A total of 93 restorative units had a follow-up of between 24 and 60 months, and 61 restoration units had a follow-up of between 12 and 24 months. A total of 7 complications were encountered (14.58% of cases; 95.45% per prosthetic unit). The technical complication rate was 2.08% (one case of minor chipping in one prosthetic unit); regarding the mechanical complications, four decementations (8.33% of the cases) and two screw loosening (4.17% of the cases) were encountered.

**Conclusions:**

Considering the limitations of this study, it can be concluded that monolithic or partially veneered zirconia implant-supported restorations have a good clinical behavior during a follow-up period of up to 5 years.

## 1. Introduction

In the past, metal-ceramics have been the restorative material of choice for tooth- and implant-supported fixed prostheses [1]. Attempts to improve the esthetic properties of these materials led to the development of all-ceramics, either by combining a ceramic core and veneer or using a single monolithic block [2]. Feldspathic porcelain was introduced first; it has a high proportion of vitreous phase in its structure, resulting in good esthetic properties, but it is mechanically inadequate in some cases [2]. Later, the fracture resistance of ceramics was improved by increasing their crystalline phase content—lithium disilicates or zirconium dioxide (ZrO_2_)—which can be used as a ceramic core with feldspathic veneer or in monolithic form. Zirconium (Zr) is a periodic table metal with properties common to the group of metals in terms of resistance and optical and chemical behavior.

Restorations with a zirconia core and ceramic veneer are widely used with good clinical results for both teeth and implants [3, 4]. In addition to providing a survival similar to metal-ceramics, they have better esthetic properties. However, one of the most common complications is a minor veneer debonding or chipping, while extensive fractures of the structure are less common [3, 4]. Different strategies have been described to prevent this limitation of the material, such as changing the structural design or using different sintering methods, but chipping remains a major problem [5, 6]. In recent years, a new type of zirconia with higher translucency has appeared; this can be used as a monolithic material without ceramic veneer, thus avoiding chipping [7, 8]. Despite the decrease in fracture resistance compared with the conventional zirconia core of prosthetic structures, its mechanical behavior is superior to metal-ceramic or zirconia ceramic restorations [9, 10]. Also, one of the main advantages of this material is the simplicity of the working process, which requires less preparation time by the laboratory technician and has a lower economic cost.

However, the upper-anterior region has higher esthetic demands, so a cut-back technique can be used on the buccal surface of the restorations to veneer feldspathic porcelain in this region. Even after adding this ceramic layer, the risk of chipping is lower, since it is only placed in nonfunctional areas [11].

Additionally, the increased use of computer-aided design and computer-aided manufacturing (CAD-CAM) technology has also improved the cost-benefit ratio. Another important factor when choosing this material is that it causes less wear on the opposing dentition than any other suitable restorative material [12, 13]. However, at present, there are not many clinical studies with a long follow-up period assessing the reliability of such material. Therefore, the aim of the present retrospective clinical study was to analyze the complications of implant-supported crowns, bridges, and full arches of monolithic or partially veneered zirconia.

The null hypothesis tested was that implant-supported crowns, bridges, and full-arches made of monolithic or partially veneered zirconia have high success rates (over 90%).

## 2. Materials and Methods

### 2.1. Data Collection

For this study, patients who consented to receive single crowns, bridges, and full-arch prostheses of monolithic or partially veneered zirconia from 2013 to May 2019 were reviewed. Patients of all ages and with different prosthetic needs at two different private clinics located in Bilbao and Vitoria (Spain) participated in the study (Table 1).

Implant placement and prosthetic protocols were carried out in the same clinical way by two skilled operators (MD and DC). Patients who underwent treatments with implant-supported prostheses ranging from single crowns to fixed partial dentures and full arches were included. Participant selection was conducted by reviewing the data of the clinic history of the patient and including those cases restored with monolithic zirconia and buccal ceramic-veneered zirconia restorations. The following data were considered: age, sex, number of prosthetic units, crowns or bridges, number of abutments and pontics, cantilever units, anterior or posterior, use of monolithic or partially veneered zirconia, zirconia trademark, use of an occlusal splint, time in mouth from placement to the last examination, mechanical and technical complications (screw loosening, decementation of the restoration, chipping, or fractures of the framework), and its solutions. The exclusion criteria were as follows: teeth-supported prosthesis, peri-implantitis in some of the implants that supported the prosthesis, implant-supported restorations made of any material other than monolithic or buccal ceramic-veneered zirconia, and restorations which had not been cement-screw-retained.

### 2.2. Clinical Procedures

Tapered bone and tissue level Klockner implants were inserted (Soadco S.L., Escaldes Engordany, Andorra). The implant position was based on a previous prosthetic-guided planning and an exhaustive clinical and radiologic examination. All implant-supported restorations were planned to be hybrid cement-screw-retained. In all the cases, cone beam computed tomography (CBCT) images in combination with three-dimensional (3D) planning software were used (Carestream Dental LLC, Atlanta, USA). All implants were placed with an open flap procedure and following a drilling protocol according to the recommendations of the manufacturer.

A healing time of 8 weeks was applied. If guided bone regeneration procedures were carried out simultaneously to the implant placement, or primary stability values were not enough (insertion torque < 35 Ncm^2^ or ISQ < 65), a healing time of 4 up to 6 months was maintained.

Once the implant was osseointegrated (ISQ values ≥ 70), prosthetic treatments were performed. The standard clinical procedure for placement of implant-supported prostheses with the one-step pick-up technique and with individual tray was applied. The obtained casts were scanned; next, the design was developed using the Trios software (3Shape Dental System, Copenaghue, Denmark) and later milled in acrylic material (Degos Dental GmbH, Regenstauf, Deutschland). These preliminary restorations served to check esthetics and occlusion. Then, the test result was sent back to the dental laboratory technician for rescanning and virtually superimposing onto the previous design using the same software (3Shape Dental System, Copenaghue, Denmark).

Finally, the final structure was milled in monolithic zirconia (Zahn Dental Labs, Melville, USA) according to the performed clinical modifications with a minimum thickness of 0.5 mm. If feldspathic porcelain would be added on the buccal side, a last intraoral test was achieved with the zirconia milled restoration with the buccal cut-back applied, and finally, it was sent back to the laboratory for finishing. All reconstructions were fabricated by the same dental technician in a private laboratory.

Before placing the restoration, a try-in occlusion check was performed. If any adjustment was done, restoration was sent back to the laboratory for polishing. For single crowns, hybrid cement-screw retention with antirotational cementing abutments with at least 2 mm of gingival height was used. In bridges, and full arches, nonengaging titanium bases with 1 mm of gingival height and 3.5 mm of cementing surface (Soadco S.L., Escaldes Engordany, Andorra) were used for hybrid cement-screw-retained restorations.

The crowns were cemented on the abutment outside of the mouth with a Maxcem Elite™ Self-Etch, Self-Adhesive Resin Dental Cement (KerrHawe, Bioggio, Switzerland). Then, in all cases, restorations were screwed into the implant at 30 N/cm^2^ according to the manufacturer's recommendations. The screw access hole was closed with flowable composite (Clearfil Majesty, Kuraray Europe, Hattersheim am Main, Deutschland).

**Figure 1:**
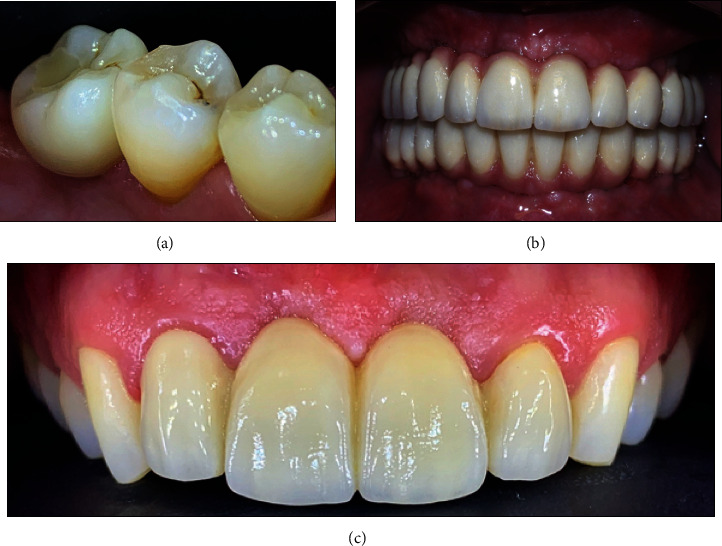
(a) Example of a single monolithic zirconia crown in 4.6. (b) Double full-arch monolithic zirconia rehabilitation. (c) 1.2-2.2 FDP made of monolithic zirconia with buccal porcelain veneered.

Patients were followed up at one month, 6 months, and finally each year except in cases requiring more attention, for example, patients with periodontal diseases who need shorter periodic follow-ups (Figure 1).

In this routine recall sessions, clinical and radiographic examination with bitewings and periapical radiographs was performed, and mechanical (decementations or screw loosening) and/or technical complications (chipping or fractures) occurred were written in the clinic history.

Due to sample heterogeneity and the impossibility of performing analytical statistics, descriptive statistics were performed and all data were collected in an Excel file, and statistical analysis was performed with computerized software (STATA/SE version 13.1, Stata Corporation).

## 3. Results

The study analyzed 154 restorations, including 34 pontics and 8 cantilevers (82 monolithic zirconia and 72 partially veneered zirconia) in 48 patients (26 men and 22 women) between the ages of 20 and 83 (mean age 56.3 years) (Table 1), yielding 65 cases and a total of 112 implants. Follow-up periods varied from 12 months to 60 months.

Of these restorations, 80 were posterior, 16 anterior, and 58 full-arch. An overview of all included zirconia implant-supported restorations is given in Table 2.

The follow-up period ranged from 24 to 60 months for 93 prosthetic units and 12 to 24 months for 61 prosthetic units. A total of 7 complications occurred. Five of the complications occurred in the group with a follow-up from 24 to 60 months (94.62% of success rate per restoration and 76.19% per case), and the remaining two complications belonged to the group with a follow-up from 12 to 24 months (96.72% of success rate per restoration and 92.59% per case). There was a single case of minor chipping that occurred in one prosthetic unit out of all the placed with partially veneered zirconia, which was resolved by polishing the ceramic surface, as it was only a minor chipping without exposing the zirconia core.

Regarding mechanical complications, four decementations occurred, which were resolved by recementation; the screw loosened in two implant-supported splinted restorations, which were fixed by retightening the screws at 30 N/cm^2^. All these complications are detailed in Table 3. All the success rates are shown in Table 4.

## 4. Discussion

This study investigated the complications of implant-supported crowns, bridges, and full arches of monolithic or partially veneered zirconia. The null hypothesis tested must be accepted for single crowns (anterior and posterior) and for posterior crowns when this group is analyzed in ratios per case.

This success rate is consistent with clinical studies such as that of Degidi et al., with 88.2% success rates in restorations placed on implants with conical abutments [14]. However, other similar clinical study related 98.4% of survival rate without any mechanical or technical complication [15]. The present study has a smaller sample size and similar follow-up periods than the first study and a longer follow-up period and a larger sample size than the second study cited. Moscovitch published a retrospective study with 600 implant-supported prosthetic units of monolithic or partially veneered zirconia with up to 68 months of follow-up and reported that one cement-retained restoration was lost to zirconia abutment fracture and replaced with a single screw-retained implant-supported zirconia restoration with a titanium base (99.83% success rate per prosthetic unit up to 68 months) [16]. The results in terms of the success of the present study agree with the results exhibited by Degidi et al. However, they found the wear of the occlusal surface as the main observed complication; on the other hand, they did not report any debonding of the restoration, because a cone-in-cone connection without cement was used. Other prospective studies in the literature reported success rates ranging between 91.1% and 98.5% and follow-up periods from 12 to 41 months [17, 18]. Regarding full-arch rehabilitations, one systematic review reported a success rate of 83.9% [19]. In this study, there were complications in 2/5 of full-arch implant-supported rehabilitations placed, resulting in 60% complication-free total cases. When considering the number of prosthetic units included in these full rehabilitations, the success rate changed to 81.03%.

In this study, only 4 of 49 patients that were rehabilitated with implant-supported FDPs used an occlusal splint. An occlusal splint was prescribed only in patients with bruxism and various joint and/or muscle disorders but not as a general measure to prevent restoration fracture.

Most studies published on zirconia describe ceramic-veneered restorations. The most commonly reported technical problem in these cases was a minor ceramic delamination or chipping. Nevertheless, it should be noted this chipping rate is very similar to that reported in the latest published reviews comparing this material with implant-supported metal-ceramic crowns [4]. This remains a significant problem, especially in bridges [3]. It almost disappeared after high translucency zirconia became available and enabled monolithic restorations with good esthetic results.

In all of the studied cases in this research, the restorative material in contact with opposing teeth was monolithic zirconia, since the feldspathic ceramic was only placed on the buccal surface of the upper teeth. The opposing teeth characteristics (natural teeth, restored teeth, or implants) were not selected as exclusion criteria to choose the final sample of the study, and this could be a limitation. However, when a prosthetic complication was detected, the opposing teeth were registered. In this sense, only one technical complication occurred—one case of minor chipping in a buccal feldspathic porcelain-veneered crown (tooth 1.1) on an upper full-arch implant-supported rehabilitation. In this case, the antagonist was a natural teeth arch without any restoration and with a mutually protected occlusal scheme with anterior guidance.

Regarding decementation and screw loosening, multiple factors could have affected: type of cement employed, size of the cementable abutment, passive fit of the restoration, and occlusal forces [20–22]. Nevertheless, all the decemented cases had a cantilever as a common factor (three of them were distal and the other one mesial), and this could be a possible cause of excessive load and leverage.

In respect of screw loosening, both cases observed were splinted posterior restorations. In theory, this could make the screw loosening more difficult. However, the higher difficulty to achieve passive fit in FDPs is well known. Hence, the lack of passive fit could have been the cause of this complication. However, a new screw loosening was not observed after retightening in successive recall sessions.

Another property of monolithic zirconia is its lower abrasion rate of the opposing tooth compared with other ceramics [12, 13]. In this regard, wear of the opposing dentition was not observed in any case at the follow-up sessions. Moreover, zirconia is highly biocompatible with adjacent tissues and presents lower bacterial adhesion than other restorative materials, resulting in a good response of periodontal and peri-implant soft tissues [23, 24]. In relation to this, no peri-implant disease was observed in any of the implant-supported restorations studied.

Furthermore, one medium- or long-term problem of monolithic zirconia is its low-temperature degradation (LTD) related to hydrothermal aging; this could cause the material to transform spontaneously from the metastable tetragonal phase to the monolithic phase. This change could affect the mechanical properties of zirconia. This initial phase transformation would facilitate that of adjacent particles because of the increase in the associated volume, thereby increasing stress in these grains and causing microcracking. Microcracks would create a path for water to penetrate the ceramic. The mechanism by which oral environmental factors (saliva, acids, temperature, moisture, and stress) affect the transformation rate is not yet clear [25]. Despite the effects of hydrothermal aging, the mechanical properties of this material are still superior to metal-ceramics and zirconia-ceramics [26, 27]. Nevertheless, it is mandatory to avoid grinding of the surface of the monolithic zirconia restorations to prevent the formation of microcracks. In this sense, it is recommended to apply an optimal prosthetic planning and a try-in with a PMMA material that allows to make all necessary esthetic and occlusal adjustments in this trial. If minimal occlusal adjustments were made in the definitive restoration try-in, it would be sent back to the technician for further surface polishing.

The retrospective nature of the present study and the different observation periods are its main limitation.

## 5. Conclusions

Considering the limitations of this study, but taking into account the use of a high number of restorations, long follow-up, and use of the same working protocols, we could affirm that monolithic or partially veneered zirconia is a material with good clinical behavior on implant-supported restorations in follow-up periods of up to 5 years. However, although many *in vitro* studies have been conducted in this field, a greater number of clinical studies are needed with a longer follow-up period to confirm the results of the present study.

## Figures and Tables

**Table 1:** Distribution of the restorations per patient's gender and age.

Age	Men	Women	Number of restorations
20s	2	0	3
30s	0	2	2
40s	4	4	14
50s	8	10	57
60s	10	3	51
70s	2	2	15
80s	0	1	12
Total	26	22	154

**Table 2:** Zirconia implant-supported restorations included.

Prosthetic restoration	Area	No. of cases	No. of restorations	No. of implants	No. of pontics and cantilevers	Monolithic	Partially veneered	Total
Single crowns	Posterior	35	35	35	-	33	2	39
	Anterior	4	4	4	-	0	4	
Fixed dental prostheses	Posterior	18	45	38	7	35	10	57
	Anterior	3	12	8	4	0	12	
Full-arch restoration	-	5	58	27	31	14	44	58
Total		65	154	112	42	82	72	154

**Table 3:** Complications encountered during follow-ups.

	Minor chipping	Decementation				Screw loosening	
Age	69	72	56	59	68	47	74
Gender	W	W	M	W	M	W	M
Affected teeth	1.1	1.4-2.5	2.4-2.5	1.6-1.4	2.4-2.6	3.6-3.7	4.6-4.7
Prosthetic design	Full arch	Full arch	FDP	FDP	FDP	FDP	FDP
Abutments/pontics	5/6	4/5	1/1	2/1	2/1	2/0	2/0
Cantilever	0	0	1 (mesial)	1 (distal)	1 (distal)	0	0
Buccal ceramic veneering	Yes	Yes	No	No	No	No	No
Occlusal splint	No	No	No	No	No	No	No
Complication time (months)	12	24	11	9	18	12	14
Follow-up time (months)	36	42	56	42	42	16	23
Solution	Polishing ceramic surface	Recementation				Retightening	

**Table 4:** Success rates per case and per restoration in all the prosthetic modalities studied.

	Success rate per case			Success rate per restoration		
	Total	Anterior	Posterior	Total	Anterior	Posterior
Single crown	100%	100%	100%	100%	100%	100%
FDP	76.19%	100%	72.22%	82.46%	100%	77.78%
Full arch	60%	-	-	81.03%	-	-
Total	89.23%	100%	90.57%	86.36%	100%	88.89%

## Data Availability

The data used to support the findings of this study are available from the corresponding author upon request.
